# Media Reports on COVID-19 Vaccinations: A Study of Topic Modeling in South Korea

**DOI:** 10.3390/vaccines10122166

**Published:** 2022-12-16

**Authors:** Keumseok Koh, Seunghyeon Lee, Sangdon Park, Jaewoo Lee

**Affiliations:** 1 Department of Geography, The University of Hong Kong, Room 10.31, 10F, The Jockey Club Tower, Pokfulam RD, Hong Kong, China; 2 Department of Industrial Security, Chung-Ang University, Heukseok-ro 84, Dongjak-gu, Seoul 06974, Republic of Korea; 3 AlphaSights Ltd., WeWork Eulji-ro 7th Floor 343, Samil-Daero, Jung-gu, Seoul 04538, Republic of Korea

**Keywords:** Coronavirus disease 2019 (COVID-19), vaccination, latent Dirichlet allocation (LDA) topic model, public health communication, media, Republic of Korea

## Abstract

Early successes in controlling the COVID-19 pandemic have prevented Republic of Korea from implementing a prompt, large-scale vaccine rollout to the public. The influence of traditional media on public opinion remains critical and substantial in Republic of Korea, and there have been heated debates about vaccination in traditional media reports in Korea. Effective and efficient public health communication is integral in managing public health challenges. This study explored media reports on the COVID-19 vaccines during the pandemic in Republic of Korea. 12,399 media news reports from May 2020 to September 2021 were collected. An LDA topic model was applied in order to analyze and compare the topics drawn from each study phase using words from the unstructured text data. Although media reports from before the national vaccination implementation focused on the development and rollout of COVID-19 vaccines, diverse topics were reported without any overlap. After the vaccination rollout, the biggest concern was the side effects of the COVID-19 vaccine. In sum, Republic of Korea’s major media outlets reported on diverse topics rather than generating a common discourse about topics related to COVID-19 vaccination.

## 1. Introduction

The coronavirus disease 2019 (COVID-19) pandemic has affected the world by causing hundreds of millions of infected cases and suspending social and economic activities. Since the outbreak, several COVID-19 vaccines have been developed to build immunity. However, hesitancies regarding these vaccines have increased, both in developing and developed countries, often shaped by misinformation and conspiracy theories that embody pseudo-scientific and anti-Western narratives [1,2]. Vaccine hesitancy is a critical barrier to achieving herd immunity, which is needed to end the COVID-19 pandemic. It is, thus, necessary to examine the information and discourses among the general public regarding this topic in order to overcome this vaccine hesitancy [2].

Topic modeling is a machine learning technique that analyzes underlying topics in a set of documents as well as the degree of affinities that exist between these topics and the relevant documents [3]. Any form of unstructured text can be analyzed to identify all of the corresponding topics by selecting items from baskets of words that are closely related to a given topic. This process is repeated until all of the baskets have a designated topic in the form of the most probable distribution of words [4]. Latent Dirichlet allocation (LDA), developed by Blei, Ng, and Jordan [5], is the most commonly used topic modeling method. It involves extracting the overall representation of a given corpus by heuristically analyzing any topics hidden within the text and then identifying any content-related clusters [6]. During this process, the LDA algorithm identifies and groups word-based patterns in order to detect the underlying topics that are not directly visible in a set of documents. Based on this, topic modeling and LDA can be used in qualitative studies to discern media biases and ideological discourses via quantitative approaches [3].

Topic modeling and LDA have been applied to various research topics, ranging from identifying trends in academic fields to sentiment analyses in social media [4,7,8,9]. From such applications, major topics have been extracted from large amounts of text data, with major agendas and societal changes being recognized. Topic modeling has also been used in various studies, including literature reviews [10], research on online support groups [11], and studies on Twitter [12].

Notably, LDA modeling has been widely used to examine numerous topics related to the COVID-19 pandemic [13,14,15,16]. A handful of studies have analyzed data obtained from both Twitter and academic papers to understand COVID-19-related discourse and the way it has changed over time [13,14]. For instance, one study used LDA modeling to explain the role of the leading media companies during the pandemic and observe changes in the flow of topics expressed in articles [15]. Likewise, another study used LDA modeling to identify and examine COVID-19-related trends in Facebook discussions [16]. A challenge to using social media data, however, comes from how well a social media platform can represent the total population. For example, Twitter was ranked 6th among social media platforms operating in Republic of Korea in 2021 [17].

In contrast, few studies have fully explored the COVID-19 discourse developed by traditional media outlets (i.e., newspapers and broadcasting companies) despite their significance as key information providers in society. Despite the recent surge in the use of social media, traditional media (often called legacy media) continues to significantly shape public opinion and discourse [18]. There are more than 5000 newspapers and 48 broadcasting outlets operating in Republic of Korea [19]. While online news consumed through the Internet on mobile devices has replaced traditional media usage, many people still use traditional media to obtain news and information on current affairs [20]. A recent media usage survey in 2020 in Korea found that the national broadcasting company Korean Broadcasting System (KBS) and Munhwa Broadcasting Corporation (MBC), a partially state-owned broadcasting company, were among the most influential media outlets for the formation of public discourse in Korea [21].

Therefore, the purpose of this study is to fill this gap by employing LDA to explore the corresponding discourse in legacy media reports on COVID-19 vaccinations in Republic of Korea. Two sets of research questions set to achieve the goal of this study include

- (Q1) Can we observe any topic changes in media reports on COVID-19 vaccination in Korea across time?
- (Q2) Were the media reports on COVID-19 vaccination in Korea informative to the public? Did the legacy media outlets in Korea focus on the topics directly related to COVID-19 vaccination or become distracted with other topics rather than public health?

## 2. Materials and Methods

### 2.1. Study Period

The Korean Disease Control and Prevention Agency (KDCA) officially announced its national COVID-19 vaccine rollout plan on 18 December 2020. The vaccination rollout then officially started on 26 February 2021, for priority groups, including medical staff and older adults aged over 80 years. The vaccine rollout was extended to the general public on 1 April 2021.

**Figure 1:**
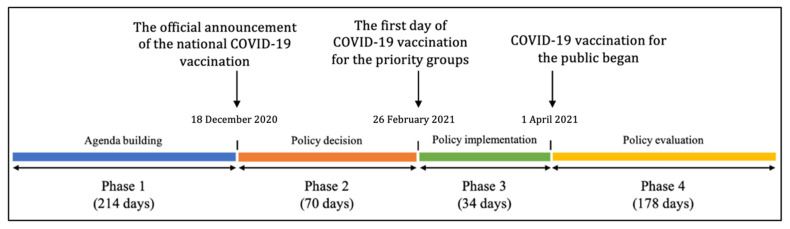
COVID-19 phases in the study period.

As illustrated in Figure 1, the study period was from 18 May 2020 to 25 September 2021. Modifying Howlett and Ramesh’s policy-making model [22], we divided this period into four phases. Phase 1 included 214 days (i.e., almost the same duration as Phase 3 to Phase 4), from 18 May 2020 to 17 December 2020, and was considered “the agenda-building period”. Phase 2 lasted for 70 days, from 18 December 2020 to 25 February 2021, and was designated as “the policy decision stage”. Phase 3 lasted 34 days, from 26 February to 31 March 2021, and was known as “the policy implementation stage”. Finally, Phase 4 comprised the 178 days from 1 April to 25 September 2021 (the cut-off date of our data collection period) and was considered “the policy evaluation stage”. Since the division of the study period was based on actual events of COVID-19 vaccination policy development in Korea, the duration of each phase varied.

These timeframes were set to examine and explore the overall development of public discourse, as they allowed for the analysis of diverse news articles that could either reinforce or reduce the public’s hesitancy toward vaccines in response to changing external situations. For each phase, any news data containing the keyword “COVID-19 vaccine” were collected and analyzed using topic modeling in order to understand the relationships between different topics as well as to identify underlying reporting trends during each phase. 

### 2.2. Data Collection

We collected news data from Big Kinds, a web-based platform that collects news articles from various sources, including newspaper companies, broadcasting companies, and news agencies in Korea [23]. The Big Kinds platform is operated by the Korea Press Foundation, which is a non-profit organization whose members include major news media companies. The platform helps users easily conduct natural language processing by extracting keywords from a large volume of news articles.

The data selected for this study were obtained from 17 leading media companies (i.e., 13 newspaper companies and 4 broadcasting companies), which have generally been perceived as the most influential, long-established media sources in Korea [24].

In total, 12,399 news articles were included in this study’s analysis. A total of 3646 articles were extracted during Phase 1, 2783 were identified during Phase 2, 1176 were obtained during Phase 3, and 4794 were found during Phase 4. Table 1 summarizes the number of news articles reported by media companies. These news articles were produced by 13 newspaper companies—Chosun Ilbo (CSIB), Donga Ilbo (DAIB), Hankook Ilbo (HKIB), Hankyoreh (HKR), Joongang Ilbo (JAIB), Korea Economic Daily (KED), Kyunghyang Shinmun (KHSM), Kukmin Ilbo (KMIB), Maeil Business Newspaper (MBN), Munhwa Ilbo (MHIB), Money Today (MT), Segye Ilbo (SGIB), Seoul Shinmun (SLSM)—and four broadcasting companies—KBS, MBC, Seoul Broadcasting System (SBS), and Yonhap Television News (YTN).

### 2.3. Data Processing

**Figure 2:**
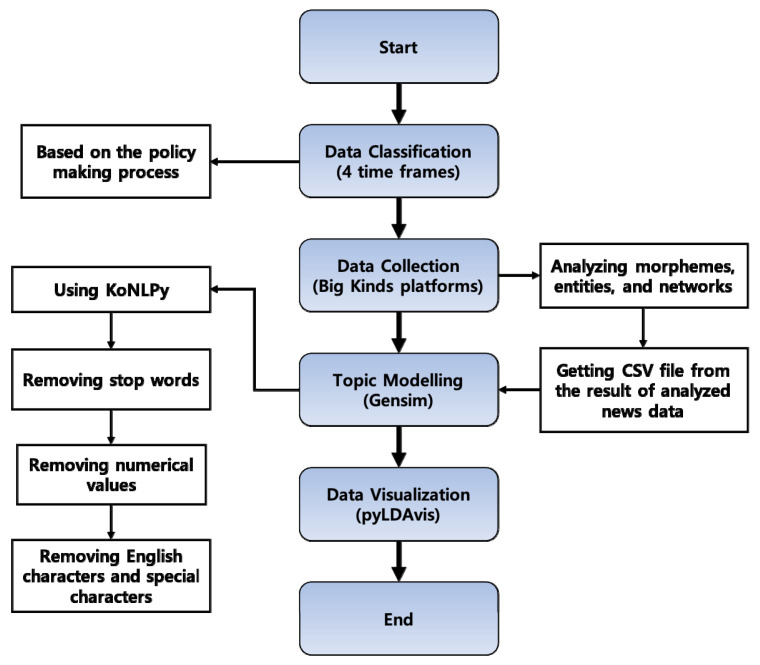
The study procedure for big data analysis on COVID-19 vaccination.

A flowchart of our research process is illustrated in Figure 2. First, we entered the search keywords “COVID-19 vaccine” and periods into the Big Kinds platform as the input. The platform then collected any corresponding news articles and analyzed their morphemes, entities, and networks. Next, it produced a CSV file containing the analyzed news data after completing all of the required processes for analyzing morphemes, entities, and networks. Then, we deleted any numerical values (e.g., 0–9), English letters, and special characters (e.g., ‘@’, ‘?’, ‘.’, and ‘!’) within the CSV file using KoNLPy, a Korean language parsing Python library [25]. We utilized the stemming and lemmatization feature of KoNLPy to convert the collected words into a common root or lemma. Next, all the data were tokenized and vectorized such that each word had a unique weight value according to its frequency. In this step, Gensim [26] was applied, which is a major topic modeling analysis tool and is a commonly used Python software program for text and language analyses. Initially, a document-term matrix was created by Gensim’s Dictionary() function. Then, the dictionary was converted into a bag of words representing weights through Gensim’s doc2bow() function.

We used LDA topic modeling [5] to examine latent topics, and topics were extracted considering the weight of the subject derived from the text set, which was identified as a probability distribution of vectors. We used random seed in Gensim analysis to prevent the same inputs from generating different conclusions.

**Figure 3:**
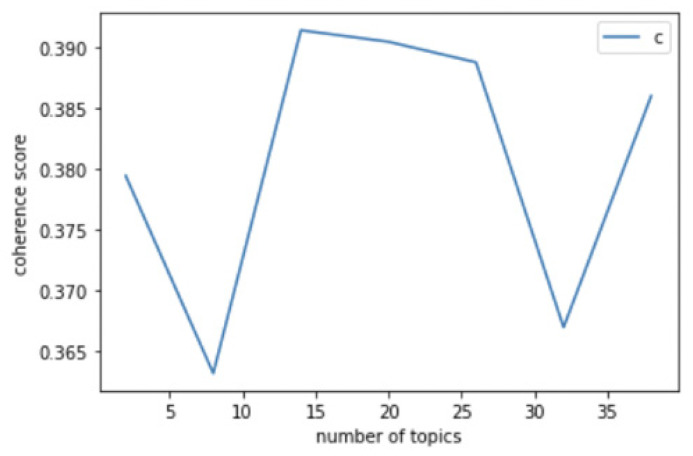
Coherence scores used to select the optimal number of topics.

To evaluate the optimal number of topics selected in the analysis, we used the coherence score model [27]. Figure 3 presents an example of the coherence scores obtained using Gensim’s coherence score module. This module quantifies the degree of semantic similarity according to the number of topics (*x*-axis). Therefore, the most appropriate number of topics for a given analysis is that with the highest coherence score. In addition to the number of topics, we needed to unify the number of repeated samplings and keywords for each topic. For this research, the number for the repeated sampling section was adjusted to 500, which is the conventional value used in other topic modeling research. The number of topics for each phase was set to 14, based on the coherence score of the entire period. After selection, topics with unrelated or meaningless keywords were filtered out in order to obtain more relevant results for the final analysis. We also provided the entire analysis result without filtering in [28].

**Figure 4:**
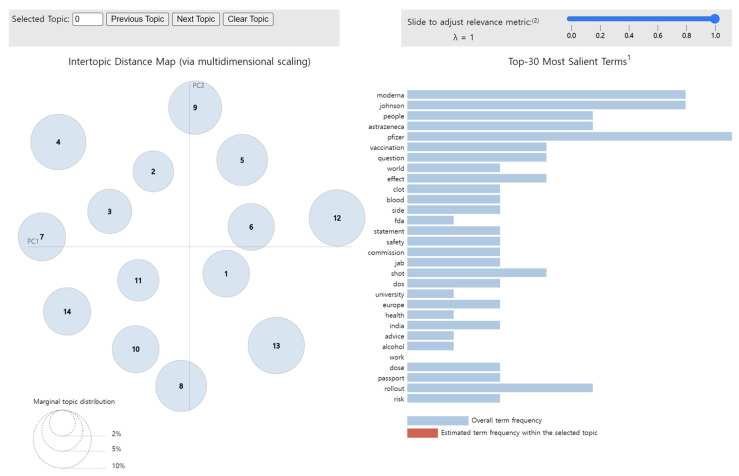
Bubble chart and bar graph for data visualization with LDA topic modeling.

To visualize the LDA results for the news articles, we used pyLDAvis [29], a Python library that draws a circle for each topic group during the modeling process. As shown in Figure 4, the distance between circles correlates with the distinctiveness of each topic, indicating the level of discriminant validity between different topics. For example, overlapping circles and circles with very little distance between them illustrate topics that possess similar content, meaning that they have low discriminant validity. Additionally, the size of the circles reflects the proportion of each topic within the total dataset obtained from the modeling process.

Therefore, this visualization through pyLDAvis enables the observation of the distribution of various topics as well as how the proportion of certain keywords in a given topic appears in others. Furthermore, when interpreting the results of this chart, lambda (λ) is adjusted to derive an accurate and detailed theme for each topic. If λ approaches 1, then the keywords are selected using the frequency measure. Otherwise, as it reaches 0, keywords that could be differentiated from other topics—that is, distinctive words—are selected, which impacts the relevance between the words contained in a given topic [30]. Consequently, to infer a valid topic from the keywords, it is necessary to derive keywords by setting an optimal value of λ.

For the data analysis, we used Google Colaboratory, a free cloud computing platform for deep learning applications [31]. In the platform, we used a virtual machine with a CPU power equivalent to the Intel Xeon 2.2 GHz with 13 GB RAM.

## 3. Results

### 3.1. Phase 1 (18 May 2020–17 December 2020)

**Figure 5:**
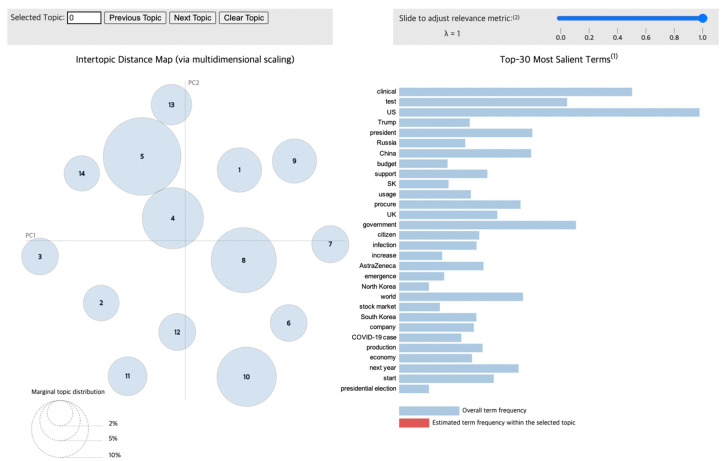
Topic visualization of Phase 1.

The topics obtained from Phase 1 are presented in Table 2, and their visualizations are shown in Figure 5. In Table 2, the prevalence of a topic (proportional to the circle size of the topic in Figure 5) indicates the frequency with which the topic is discussed in a given phase, which implies the relative importance of the topic [29]. The ranking (0–19) indicates the proportion each word constitutes within its topic. Specifically, words with higher rankings reflect a greater portion of ongoing public discourse during the designated time frame than those with lower rankings. Accordingly, the word order of the top 10 words is presented in Table 2. For example, the article “AstraZeneca launches phase 3 clinical trial for COVID-19 antibody treatment” would be converted into a common root or lemma, then each word would belong to a particular topic, especially topic 5, according to the similarity between the words in the article and the topic. As each quadrant was grouped into a similar theme, the interpretation of these results was also based on the resulting quadrants.

Highly ranked words from Quadrant I included “world”, “conference”, “SK”, “Korea”, “medicine”, and “cooperation”, which reflect countries’ responses to the pandemic by engaging in preparatory measures for responding to COVID-19, both domestically and internationally. Additionally, Quadrant II includes “test”, “result”, “usage”, “provision”, “US”, “Russia”, and “China”, which reflect the fact that certain countries were in the process of developing vaccines at this time. As Phase 1 occurred when COVID-19 vaccines were actively being developed, the progress of each country herein, including in the forms of clinical trials and results, was derived as a key topic (Figure 5).

Topic 5, which accounts for the largest proportion of topics in Phase 1, shows that a specific discourse was formed based on the results of the vaccines’ clinical trials. Topics 4 and 13, which are located close to Topic 5, outline the situation in which countries around the world were trying to secure vaccines and discussing plans for rapid vaccination procedures. In addition, both domestic and international social and economic changes arose due to imperfect vaccines and the steadily increasing number of COVID-19 cases. Domestically, financial support was provided to assist people in Korea, and certain social regulations were imposed to combat increasing infection rates. For example, social distancing levels were adjusted through terms like “COVID-19 cases”, “quarantine”, and “level”. Words that describe the political context of this pandemic include “North Korea”, “minister”, and “government”. Meanwhile, the international issues surrounding the vaccine supply chain were reflected by the fact that terms like “company”, “US”, “hacking”, and China” were also highly ranked, which highlights the progress in countries that were developing the vaccine.

The topics from Phase 1 reflect the fact that public discourse focused on developing vaccines and the changes in society caused by their development. This interpretation is supported by words that indicate both the results and the effects of the vaccines, as well as relative social issues that reflect the situation prior to the vaccines’ introduction in earnest.

### 3.2. Phase 2 (18 December 2020–25 February 2021)

**Figure 6:**
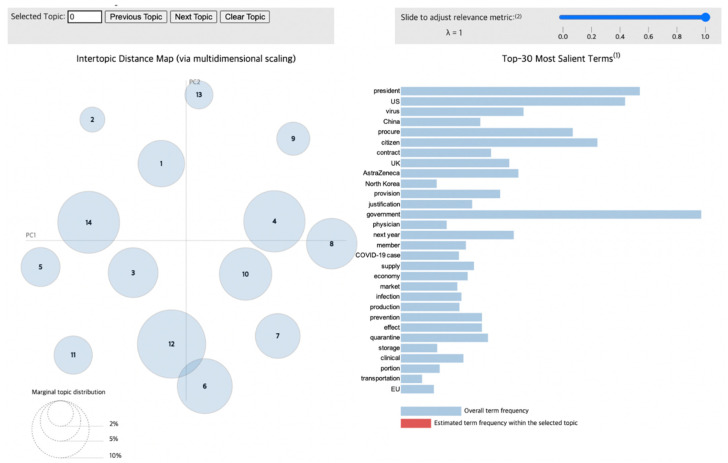
Topic visualization of Phase 2.

The topics obtained from Phase 2 are listed in Table 3, and their visualizations are shown in Figure 6. The most pronounced contrast with Phase 1 can be ascribed to the cessation of vaccine development via clinical trials and the transition toward the active vaccination process as indicated by words like “procure”, “contract”, and “subject”. This trend reflects the global period of producing and securing vaccines for the start of international vaccination drives.

In Quadrant I—which includes Topics 1 and 14, which account for a relatively large proportion when compared to other topics—words like “procure”, “contract”, “provision”, “start”, and “production” were highly ranked. This implies that the international production of vaccines started in this period and that the government struggled to obtain sufficient doses. In addition, words like “transport”, “temperature”, and “storage” were found within a completely different topic, suggesting that the storage and transport of vaccines represented a separate issue in each country.

In Phase 2, a topic that had not been observed before was derived. The words in Topic 6 are based on the results derived from Lambda 1 (λ = 1), which reflects the main content of the clinical trials. For example, the article “Pfizer vaccine is 94% effective in preventing... 1.2 million surveys in Israel” focused on the universal effects of vaccines. However, if lambda is adjusted to 0.6 (λ = 0.6), more detailed terms like “mutation”, ”infection”, “UK”, and “discovery” are derived, reflecting the situation in which a new variant was discovered in the UK and then transmitted internationally. Entire topic modeling results for all the topics with λ = 0.6 can be found in supplement [28]. Notably, in this topic, the circles of Topics 6 and 12 overlap, unlike those of other periods. The distance between the circles indicates the correlation between the topics that they reflect. These results arose from the growing concerns about increasing infection rates, meaning that rapid vaccination was then discussed domestically. Therefore, specific groups were selected for gradational vaccination. Topics 12 and 3, which contain the terms “subject”, “medical”, “medical work”, “work”, and “KATUSA (Korean Augmentation Troops to the United States Army)”, suggest that the discourse around deciding vaccination subjects arose in accordance with the prioritized job characteristics in Korea after vaccines were secured. With the vaccination of all domestic medical workers and United States Forces Korea (USFK) members, who were given priority, domestic vaccinations then began in earnest. Meanwhile, unlike in Phase 1, new words like “allergy”, “side-effect”, and “response” emerged, indicating increased concerns about the reactions to and side effects of vaccines. Other interesting trends can be found among words that refer to cyber threats like “hacking”, “medicine”, “North Korea”, and “extortion”. These words increased in use alongside the increasing value of the vaccine, as well as North Korea’s attempts to hack the vaccine’s technology.

The initiation of the vaccination drives and the resulting social phenomena were primarily addressed in Phase 2. This phase included the topics of deciding on appropriate target groups for vaccination, rising anxiety about their side effects, and early societal responses after vaccines were introduced. Nevertheless, people throughout the world continued to be exposed to high infection rates due to novel COVID-19 variants; therefore, the necessity of developing vaccines and achieving vaccination is an ongoing concern.

### 3.3. Phase 3 (26 February 2021–31 March 2021)

**Figure 7:**
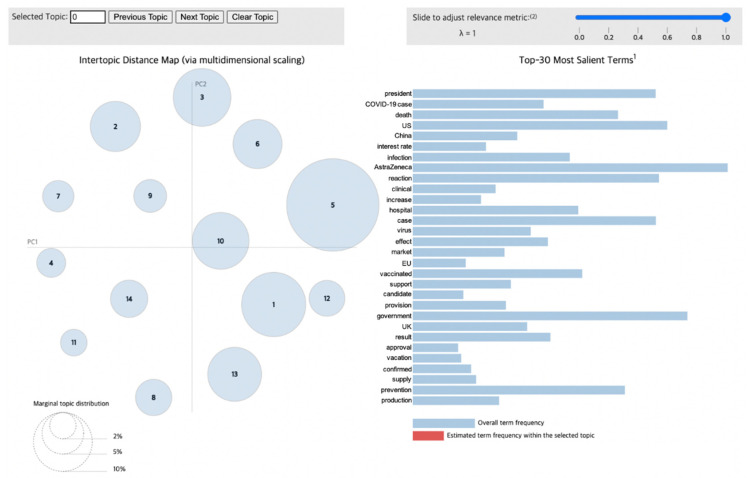
Topic visualization of Phase 3.

The topics obtained from Phase 3 are presented in Table 4, and their visualizations are shown in Figure 7. The most noticeable theme during Phase 3 comprised the topics associated with the “effects” of vaccines. This trend is reasonable, given that the vaccination rate began to increase in Korea in Phase 3. Specifically, among the 14 topics, Topic 5 featured the largest proportion, followed by Topic 10, with words like “reaction”, “death”, “symptom”, and “efficacy” being highly ranked. Furthermore, public attention was directed toward the storage and transport of vaccines, demonstrated by words like “transport”, “storage”, “temperature”, “freezer”, and “keeping”. International developments were also observed in Quadrant II. Words that refer to neighboring countries, like “China” and “Japan”, as well as those referring to influential countries around the globe, such as “the U.S”. and “Russia”, were mentioned within the international cooperation dimension. Furthermore, trends in promoting COVID-19 passports for cross-border movements among various countries were observed based on words like “passport”, “border”, “progress”, and “issuance”. In Phase 3, most of the public discourse was related to the efficacy of the vaccines, along with storage and transport guidelines. Given the increasing number of COVID-19 cases, in addition to prolonged social distancing and gathering restrictions, these topics were more widely covered across media platforms when the necessity of vaccination was clarified.

### 3.4. Phase 4 (1 April 2021–25 September 2021)

**Figure 8:**
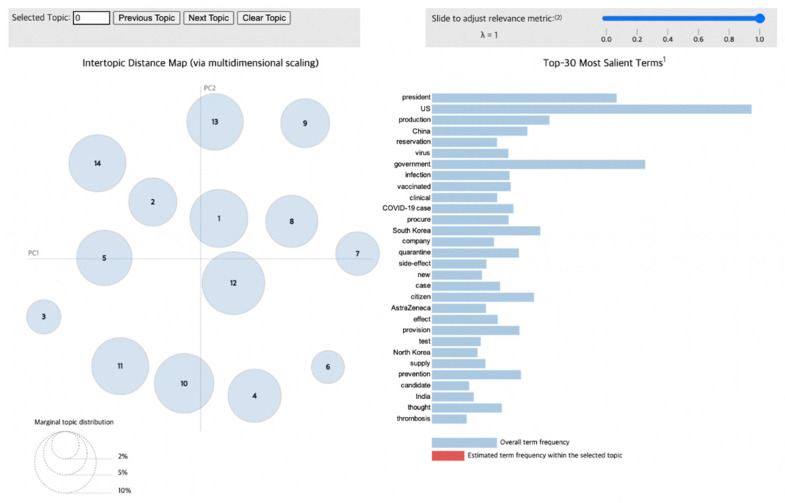
Topic visualization of Phase 4.

The topics derived during Phase 4 are presented in Table 5, and their visualizations are depicted in Figure 8. During Phase 4, the vaccination rate started to increase in Korea as vaccines were offered to additional age groups. Owing to this increase, reservations for the vaccines increased substantially. In addition, at the same time that sequential vaccinations were administered according to age groups, reservations for remaining vaccines also increased. Topic 9 reflects these situations with words such as “reservation” and “vaccinated”. Accordingly, words that reflect vaccine supply (e.g., “procure”, “supply”, and “provision”) were placed in noticeable positions.

However, as the vaccination rate increased, the concentration of public discourse on the side effects of vaccines continued. In particular, the word “thrombosis”, which was not found in the previous phase, arose within the discourse on the occurrence of new side effects. In addition, words from Topic 14 suggest that vaccine development in Korea is still underway. The fast-growing vaccination rate has also raised expectations for wider societal improvements in pandemic restrictions. In particular, the word “travel” in Topic 12 indicates that people’s expectations of traveling abroad increased. Nevertheless, a new COVID-19 variant, called Delta, arose and increased international infection rates. Domestically, the social distancing level is still being adjusted according to the infection rate. Furthermore, Korea will continue to enforce quarantine rules even during the vaccination period by preparing policies to be used during the holiday season. 

During Phase 4, vaccinations were introduced to the wider public. In this period, public discourse was formed based on vaccinations and focused on societal changes as the vaccination rate increased, whereas the efficacy of vaccines was emphasized in the previous phase. In this way, public discourse around COVID-19 vaccines has shifted toward topics like the vaccination procedure and its prospects.

## 4. Discussion

This study applied LDA topic modeling in order to analyze developments in Korean legacy media regarding the COVID-19 vaccine rollout by dividing the study period into four phases according to changes within the policy-making process. 

During Phase 1, issues related to vaccine development and clinical trials were primarily discussed in response to the ongoing global research on vaccines. Subsequently, further rollout plans for vaccination were discussed in several countries, and public interest in the introduction and procurement of vaccines increased. As expectations for vaccine development increased, cyber-attack trends aimed at blind spots in the vaccine distribution process developed. Furthermore, governmental support, as a type of social phenomenon, was introduced in consideration of the domestic COVID-19 situation and the resulting economic slowdown. At the same time, vaccines were being developed to reduce the rapidly increasing infection rates. Furthermore, the positive results of vaccine clinical trials accelerated the development of this discourse, and accordingly, several countries began to secure vaccines.

In Phase 2, discussions were held to select key targets for initial vaccination drives, with medical workers and USFK members being vaccinated first due to the relatively high importance of their occupations. This discourse developed as international concerns about the spread of infections increased because of the virus’s mutations. In other words, Phase 2 coincides with the introduction of the vaccine in Korea. Vaccination started with a few specific groups, and the target range of vaccinations in Korea gradually expanded. The increased demand for vaccines required increased efforts to achieve a sufficient supply, and national methods for transporting and storing vaccines subsequently became a key discourse feature. 

As the public’s interest in vaccines increased along with the vaccination rate, a notable topic emerged during Phase 3. As shown in the bubble chart of Phase 3, the size of the circle representing Topic 5 is the most prominent. This result reflects the growing discourse on the number of side effects as the vaccination rate increased exponentially. Regarding economic factors during this period, the financial market deteriorated as concerns over an economic slowdown caused by the ongoing COVID-19 situation grew.

Unlike in the other phases, the sizes of the derived circles during Phase 4 were relatively equal, as shown in the graph depicting its characteristics. This shows that the various extracted topics were not completely distinct. In Phase 4, vaccinations were administered across the country, starting with those aged 50 years and above. In Korea, reservations for vaccinations were required. After people of this age group and healthcare workers were vaccinated, the public’s interest focused on reservations for non-priority groups. Thus, the number of vaccinations rapidly increased, and various reactions emerged. Accordingly, the efficacy and side effects of the vaccines developed as a wider public discourse. Of note, topics related to travel within the public discourse emerged during Phase 4. Furthermore, discourses on the more positive developments of societal phenomena increased in terms of social activities, such as attending movies and entertainment performances.

In this study, newspaper and broadcasting news reports were analyzed using the keyword “COVID-19 vaccine” to determine the overall societal trends that occurred during the pandemic. The spread of COVID-19 has profoundly impacted the world, owing in part to associated social changes as shown in the media during this period. Thus, effective countermeasures to resolve issues being expressed in public discourse are strongly recommended.

In this respect, this study has several important implications. First, examining media reports through text mining in a data-driven way can be an alternative way for governments to fully understand the public’s opinions toward new policy interventions. Second, media reports may need to enhance the quality of their reports on public health challenges. For example, while a volume of media reports in Korea appropriately disseminates the latest development and discussion of COVID-19 vaccination, oftentimes less relevant information on the public health crisis was also provided to the public. For example, the topics like political disputes and international travel were heavily reported during the pandemic. Third, all the stakeholders related to public health communications, such as governments, journalists, public health professionals, and communication researchers, may need to make some guidelines or recommendations for effective public health communication under specific conditions. By so doing, more scientific and in-depth information can be extended from the governments and health professionals to the general public. Finally, future studies can explore the public’s opinions by conducting comparative studies between social media and legacy media. As mentioned above, the public opinions collected by social media may not fully represent the whole population. In contrast, legacy media are believed to go through rigorous fact-checking procedures for more objective, balanced reports. In this regard, two sources can be supplementary with each other for a full understanding of the public. 

In a nutshell, under future public health crises, key officials should actively monitor the public discourse around them so that the media can contribute more constructively to the formation of positive macroscopic discourse and socially integrative responses, with the media then developing in an appropriate and beneficial manner.

## 5. Conclusions

Korean news and broadcast media developed after the introduction of COVID-19 vaccines were analyzed using LDA topic modeling. Through this analysis, we identified changes in the broad public discourse by examining similar topics mentioned over several phases and distinctive topics that were associated only with specific phases. Moreover, topics that matched the corresponding policy-making process were extracted in each phase.

The results reveal that traditional media significantly impacted public discourse among communities impacted by social media. Thus, traditional media remains an indicator of international trends surrounding vaccinations and social policies. In addition, by reporting on and forming public discourse, the media influences health-related communication among the public. Our findings provide information for effective decisions regarding the formation of public discourse and how to prepare it by predicting social trends.

## Figures and Tables

**Table 1:** The number of articles extracted during each COVID-19 phase.

Phase 1 (18 May 2020–17 December 2020)		Phase 2 (18 December 2020–25 February 2021)		Phase 3 (26 February 2021–31 March 2021)		Phase 4 (1 April 2021–25 September 2021)	
CSIB *	703	CSIB *	556	CSIB *	286	CSIB *	1247
DAIB *	107	DAIB *	99	DAIB *	66	DAIB *	108
HKIB *	157	HKIB *	141	HKIB *	51	HKIB *	190
HKR *	76	HKR *	66	HKR *	27	HKR *	100
JAIB *	309	JAIB *	207	JAIB *	55	JAIB *	306
KED *	425	KED *	273	KED *	113	KED *	338
KHSM ^*^	88	KHSM *	63	KHSM *	17	KHSM *	66
KMIB *	165	KMIB *	96	KMIB *	51	KMIB *	111
MBN *	234	MBN *	151	MBN *	46	MBN *	196
MHIB *	50	MHIB *	37	MHIB *	9	MHIB *	44
MT *	477	MT *	253	MT *	122	MT *	585
SGIB *	158	SGIB *	122	SGIB *	63	SGIB *	295
SLSM *	183	SLSM *	168	SLSM *	64	SLSM *	211
KBS **	91	KBS **	119	KBS **	46	KBS **	225
MBC **	107	MBC **	89	MBC **	37	MBC **	173
SBS **	91	SBS **	88	SBS **	33	SBS **	209
YTN **	225	YTN **	255	YTN **	90	YTN **	390
Total	3646	Total	2783	Total	1176	Total	4794

* Newspaper companies. ** Broadcasting companies.

**Table 2:** Topics drawn from Phase 1 (18 May 2020–17 December 2020).

Topic # (Prevalence)	Subject	Words
1 (6%)	Global Responses to COVID-19	world, minister, Japan, UK, economy, summit, response, emphasis, conference, countries
4 (11.5%)	The acquisition and supply of vaccines around the world	China, procure, government, provision, US, contract, purchase, justification, next year, AstraZeneca
5 (18.5%)	Results of the vaccines’ clinical trials	clinical, test, US, result, effect, virus, AstraZeneca, candidate, progress, antibody
6 (4.1%)	Hacking the vaccine distribution network	company, US, economy, hacking, China, information, stock, market, Korea, world
8 (13%)	Status of infection and quarantine	COVID-19 cases, infection, situation, degree, quarantine, level, occurrence, patient, thought, mask
9 (6%)	The development of treatments in Korea	SK, Korea, president, medicine, cooperation, Bill Gates, support, Bioscience, CEO, production
11 (4.6%)	Stabilization of people’s livelihoods in Korea	budget, support, citizen, payment, relief fund, government, national assembly, the budget bill, next year, supplementary
13 (5.1%)	Emergency use of vaccines	US, usage, emergence, start, next year, UK, BioNTech, FDA, food, CDC
14 (3.8%)	The start of vaccination	Russia, Sputnik, world, test, clinical, president, Putin, registration, Brazil, center

**Table 3:** Topics drawn from Phase 2 (18 December 2020–25 February 2021).

Topic # (Prevalence)	Subject	Words
1 (6.6%)	Production of vaccine	China, production, US, SK, company, shot, Korea, world
2 (1.9%)	North Korea’s attempt and suspicions to hack into treatment information	North Korea, hacking, National Intelligence Service, medicine, Israel, Pope, member, extortion, information, cyber
3 (7.6%)	Vaccination of United States Forces Korea	economy, US army, USFK, world, US, government, Korea, recovery, KATUSA
5 (4.7%)	Initiation and side effects of vaccination	US, Trump, start, allergy, CDC, side-effect, people, reaction, local time
6 (9.2%)	Vaccine clinical trials and results	(λ = 1) virus, UK, effect, clinical, infection, result, test, variant, US, immune
		(λ = 0.6) virus, UK, effect, mutation, clinical practice, examination, infection, antibody, protein, discovery
10 (8.4%)	Status of vaccine acquisition in Korea	government, procure, citizen, quarantine, member, Japan, criticism, situation, comment, president
12 (14.2%)	Sequential vaccination plan in Korea	prevention, government, start, AstraZeneca, plan, subject, medical, work, hospital
13 (2.4%)	Transportation of vaccine	transport, temperature, storage, maintenance, sub-zero, freight, cold chain, distribution, Korean Air
14 (11.7%)	Plan on acquisition and supply of the vaccine	government, procure, contract, provision, next year, AstraZeneca, justification, supply, US, start

**Table 4:** Topics drawn from Phase 3 (26 February 2021–31 March 2021).

Topic # (Prevalence)	Subject	Words
2 (7.3%)	The international situation	President, US, China, summit, government, Trump, cooperation, minister, Japan, conference
5 (24.7%)	Vaccination and the occurrence of side effects	reaction, AstraZeneca, death, case, hospital, prevention, side-effect, vaccinated, symptom, occurrence
6 (7%)	Adjusting amounts of AstraZeneca in Korea and overseas countries’ interest in vaccine effects for mutant viruses	provision, EU, production, UK, supply, AstraZeneca, government, SK, export, justification
7 (2.8%)	Vaccination and consequent social change	China, Japan, Seocho, passport, border, clinical, Russia, progress, North Korea, issuance
8 (3.8%)	Domestic political issues and vaccine transportation	candidate, transport, mayor, Korean Air, election, president, citizen, thought, Seoul, member
10 (9.3%)	Vaccination and its side effect	US, effect, virus, clinical, result, infection, human, antibody, prevention, test
11 (2%)	Vaccine storage and transportation	storage, temperature, Daegu department store, transport, freezer, refrigerator, keeping, situation, underlying, distribution
14 (4%)	Transportation of vaccine	start, transport, virus, arrival, Genexine, storage, progress, center, immune, ingredient

**Table 5:** Topics drawn from Phase 4 (1 April 2021–25 September 2021).

Topic # (Prevalence)	Subject	Words
1 (8.8%)	The issue of securing domestic vaccine supplies	government, procure, president, citizen, supply, provision, portion, contract, situation, supply, and demand
2 (6.1%)	Concerns about Infection due to the holidays	Japan, vacation, information, thought, government, people, certification, offer, citizen
7 (5%)	Infection status and clinical trials of the vaccine	virus, infection, effect, antibody, result, professor, immune, prevention, people, gene
8 (7.1%)	Quarantine level in Korea	situation, quarantine, degree, government, thought, saying, COVID-19 case, possible, distance, professor
9 (6.3%)	Latest development in the vaccine rollout	reservation, vaccinated, COVID-19 case, new, test, case, region
12 (10.4%)	Debate over the Vaccine passport system	US, mask, UK, travel, Israel, Delta, human, CDC, population, infection
13 (8.4%)	Side effects of vaccines	side-effect, AZ, AstraZeneca, case, thrombosis, reaction, Janssen, hospital, occurrence, death
14 (8.6%)	Clinical trials of vaccines	production, clinical, SK, company, global, support, test, progress, Novavax, plan
